# High-Throughput and Automated Detection of HLA-B*27 Using the LabTurbo^TM^ AIO System

**DOI:** 10.3390/biomedicines11030986

**Published:** 2023-03-22

**Authors:** Yung-Che Chou, Tze-Kiong Er

**Affiliations:** 1 Division of Laboratory Medicine, Asia University Hospital, Asia University, Taichung 413, Taiwan; j8a8c8k8y8@gmail.com; 2 Department of Medical Laboratory Science and Biotechnology, Asia University, Taichung 413, Taiwan; 3 Department of Nursing, Asia University, Taichung 413, Taiwan

**Keywords:** semi-automated, fully automated, human leukocyte antigen B27

## Abstract

The adoption of an automated system can decrease the hands-on time requirements in a clinical laboratory setting. For the detection of HLA-B*27, implementing a high-throughput and fully automated system has several advantages over using manual methods. Therefore, this study aimed to evaluate automation efficiency for the detection of HLA-B*27. Peripheral blood samples were obtained from 50 participants, and DNA was isolated from these samples. A Pharmigene PG27 detection kit was used for the qualitative detection of HLA-B*27. The performances of the semi-automated and fully automated LabTurbo^TM^ AIO systems in the detection of HLA-B*27 were compared. The mean absorbance (optical density) values for the Maelstrom^TM^ 8 and LabTurbo^TM^ AIO systems were found to be 1.88 and 1.9, respectively. The housekeeping gene was amplified and quantified using a real-time PCR assay across all DNA extracts to check the quality of the extracted human DNA. The results were expressed as the cycle threshold (Ct) values for all DNA extracts from both platforms. The mean Ct values for the Roche Cobas z480 and LabTurbo^TM^ AIO systems were found to be 22.7 and 20.4, respectively. This study demonstrated that the semi-automated method and the LabTurbo^TM^ AIO system yield consistent results for the detection of HLA-B*27. However, compared to the semi-automated method, the LabTurbo^TM^ AIO system provides standardized procedures, avoids manual handling, and improves turnaround time.

## 1. Introduction

The advancement in molecular diagnostics has facilitated the genetic testing of genetic diseases as well as the identification of multiple pathogens. The operation of these tests requires extensive training in molecular biology. The implementation of laboratory automation can simplify the process of analyzing multiple specimens, thereby increasing the throughput of specimen testing, standardizing the performance of molecular testing procedures, shortening testing turnaround times, and reducing labor time and cost. In essence, automation combines laboratory robotics and analytical software. Nowadays, automation is becoming more sophisticated than simply facilitating the integration of sample extraction, as it also supports nucleic acid amplification and detection. A fully automated molecular diagnostic system is a robust and useful system that is capable of a broad variety of applications [1,2].

As molecular diagnostics is a complex process involving several procedures from nucleic acid extraction to result interpretation, technologists can only process a few clinical samples at a time. Moreover, when high-volume testing is demanded, higher manpower costs are incurred in the long run. Additionally, regular laboratory testing is highly labor-intensive and may lead to manual errors, which can result in poor patient outcomes. If the number of clinical samples to be processed by laboratories increases dramatically, productivity has to be improved. Therefore, automated equipment such as robotic liquid-handling platforms can replace manual processes in molecular diagnostics workflows. This will reduce hands-on time and improve accuracy in a clinical laboratory setting. Bioinformatic software also facilitates standardized reporting and the interpretation of laboratory test results.

Recently, Chang et al. reported that the LabTurbo^TM^ AIO (Taigen Bioscience Corp., Taipei, Taiwan) COVID-19 RNA testing kit is a feasible and reliable method for SARS-CoV-2 detection [3]. They demonstrated a sample-pooling strategy using the LabTurbo^TM^ AIO extraction system, which is combined with a real-time reverse transcription polymerase chain reaction (rRT-PCR) platform. According to them, the pooling strategy applied in the LabTurbo^TM^ AIO system resulted in zero volume loss for SARS-CoV-2 detection using rRT-PCR [4]. Chung et al. also reported that the multiplex RT-PCR implemented on the LabTurbo^TM^ AIO system offered a high-throughput detection of SARS-CoV-2 variants, influenza A/B, and respiratory syncytial virus, along with shortening the duration of the entire process from its initiation to completion [5]. Until recently, the LabTurbo^TM^ AIO system had not been used for the detection of HLA-B*27. 

The human leukocyte antigen (HLA) system is a gene complex encoding the major histocompatibility complex proteins in humans. These cell-surface proteins are in charge of the regulation of the immune system. Classic class I HLA consists of HLA-A, -B, and -C genes, whereas the main class II HLA genes are DP, DQ, and DR [6]. Studies demonstrated that HLA genetic variants are associated with systemic lupus erythematosus, rheumatoid arthritis, multiple sclerosis, type 1 diabetes mellitus, celiac disease, narcolepsy, and Sjögren’s syndrome [7,8]. HLA-B*27 is strongly associated with ankylosing spondylitis (AS), and HLA-B*27 detection is routinely applied in the diagnosis of this disease. The percentages of HLA-B*27 positivity in the general population and among AS patients are 5–10% and 90%, respectively [9]. Multiple molecular diagnostic techniques are applied for the routine typing of HLA-B*27, including PCR with sequence-specific primers, PCR with restriction fragment length polymorphism, and also PCR with sequence-based typing, flow cytometry, melting curve analysis, and real-time PCR assay [10,11,12,13,14]. Thus far, a fully automated procedure has not been applied to the detection of HLA-B*27. As mentioned above, the adoption of an automated system can decrease the hands-on time requirements in a clinical laboratory setting. For the detection of HLA-B*27, implementing a high-throughput and fully automated system has several advantages over using manual methods.

In the present study, we aimed to evaluate the efficiency of automation in the detection of HLA-B*27.

## 2. Materials and Methods

### 2.1. Study Population

The present study included a total of 50 participants who had requested HLA-B*27 typing. Out of the 50 samples that were tested, 35 (70%) were obtained from male patients while 15 (30%) were obtained from female patients. The median age of these 50 patients was 47.5 years (12–69). Peripheral blood specimens (10 mL) were collected in K_2_EDTA. One part of each sample was analyzed using a semi-automated procedure, whereas the other part was analyzed using a fully automated procedure. Ethical approval was obtained from the Institution Review Board of China Medical University Hospital (CMUH112-REC2-026) and (CMUH112-REC2-026 (AR-1)). The requirement for informed consent from each patient was waived.

### 2.2. DNA Extraction and Real-Time PCR Analysis

In this study, the Maelstrom^TM^ 8 Autostage (Taiwan Advanced Nanotech, Taoyuan City, Taiwan) was used for extracting DNA from the whole blood samples using the TANBead^®^ nucleic acid extraction kit (Taoyuan City, Taiwan), as per the manufacturer’s instructions. DNA was extracted from samples of 300 μL volume. The Maelstrom^TM^ 8 Autostage is a high-efficiency purification system driven by TANBead’s patented technology [15]. With TANBead Maelstrom^TM^ 8 Autostage, a maximum of eight samples can be processed during each run.

The LabTurbo^TM^ AIO system (LabTurbo Biotech Corporation, Taipei City, Taiwan) was used for extracting DNA from the whole blood samples using the LabTurbo Viral DNA/RNA Mini Kit (Cat. No. LVX480-500; Taigen Bioscience Corp., Taipei City, Taiwan), as per the manufacturer’s instructions. The total DNA extracted from the whole blood sample (50 µL) was a final eluate volume of 60 µL. With the LabTurbo^TM^ AIO system, a maximum of 48 samples can be processed during each run. The purity of the extracted DNA was determined using a spectrophotometer. We used the HLA-B*27 detection kit (Pharmigene, Inc., Taipei city, Taiwan) to the LabTurbo^TM^ AIO system, using specific primers and probes to detect the HLA-B*27 allele. The HLA-B*27 detection kit (Pharmigene, Inc., Taipei city, Taiwan) was handled according to the manufacturer’s instructions. The sensitivity and specificity of the HLA-B*27 detection kit were greater than 99%, which indicates the high sensitivity of major HLA-B*27 subtypes, including HLA-B*2701~2707. The procedures followed for the LabTurbo^TM^ AIO system are presented in the flow chart in the supplementary data (Figure S1).

For analysis on the LabTurbo^TM^ AIO system, each 25 μL reaction mixture contained 12.5 μL of 2 × PCR master mix, 2.5 μL of primer/probe mixture, 5 μL of RNase-free water, and 5 μL of extracted RNA. HLA-B*27 was detected using the following thermal cycling conditions: 95 °C for 10 min, 35 cycles at 95 °C for 15 s, and 71 °C for 60 s (collection of fluorescent signals at 71 °C).

For analysis on the Roche Cobas z480 system (Roche Molecular System Inc, Pleasanton, CA, USA), each 25 μL reaction mixture contained 12.5 μL of 2 × PCR master mix, 2.5 μL of primer/probe mixture, 8 μL of RNase-free water, and 2 μL of extracted RNA. HLA-B*27 was detected using the following thermal cycling conditions: 95 °C for 10 min, 35 cycles at 95 °C for 15 s, and 71 °C for 60 s (collection of fluorescent signals at 71 °C).

### 2.3. Statistical Analysis

**Figure 1:**
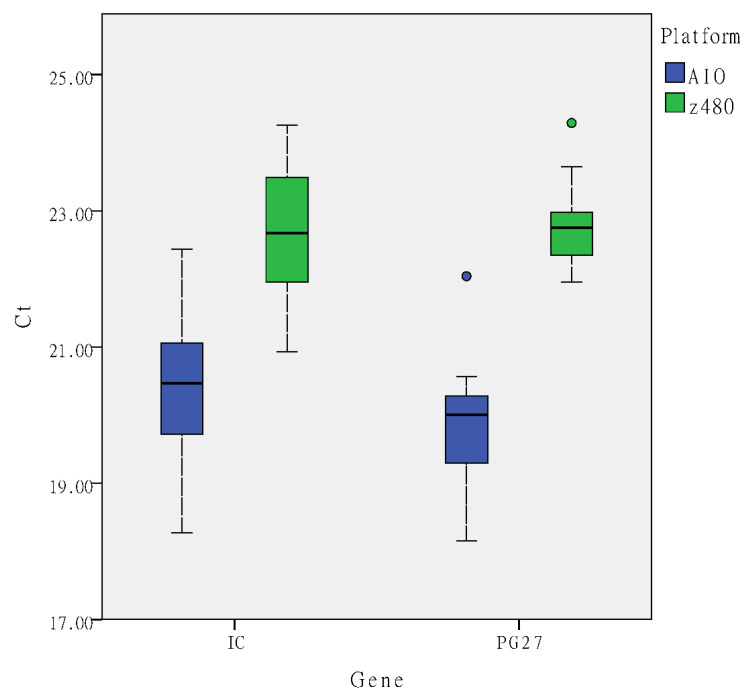
Ct value of the distribution of target genes of Maelstrom^TM^ 8 and LabTurbo^TM^ AIO system when analyzing the clinical samples. Box plots illustrate the median value and interquartile range, and whiskers represent the nearest value to 1.5 times IQR. Outliers are plotted separately. IC (internal control), PG27 (HLA-B*27).

The software used for the statistical analyses was SPSS version 22.0 (IBM Corp., Armonk, NY, USA) for Windows. The detailed results are represented in Figure 1. The box plot displays the distribution of the Ct values for the 50 samples analyzed in the present study, while the horizontal black bar represents the median Ct value. The lower and upper boxes indicate the 25th percentile to the 75th percentile.

## 3. Results

### 3.1. Semi-Automated and Fully Automated Procedures in DNA Extraction 

The mean absorbance (optical density) values for the Maelstrom^TM^ 8 and LabTurbo^TM^ AIO systems were observed to be 1.88 and 1.9, respectively. The housekeeping gene was amplified and quantified using a real-time PCR assay in all of the DNA extracts to check the quality of the extracted human DNA, and the results were expressed as the cycle threshold (Ct) values for all DNA extracts from both platforms. The mean Ct values for the Roche Cobas z480 system (Roche Molecular System Inc, Pleasanton, CA, USA) and the LabTurbo^TM^ AIO system were 22.7 and 20.4, respectively. Figure 1 presents the Ct values of the distribution of the target genes of the Maelstrom^TM^ 8 and LabTurbo^TM^ AIO systems obtained by analyzing the clinical samples.

### 3.2. Semi-Automated and Fully Automated Procedures in HLA-B*27 Detection

We validated the performance of the semi-automated and fully automated assays designed in this study for the detection of HLA-B*27. The LabTurbo^TM^ AIO system obtained a positive percent agreement of 100% as well as a negative percent agreement of 100% by using a semi-automated assay (Table 1). The LabTurbo^TM^ AIO system performed HLA-B*27 detection on 48 samples in under 200 min compared to the semi-automated system. The incorporation of a fully automated extraction and real-time PCR assay on the LabTurbo^TM^ AIO system significantly reduced the operation time to 110 min compared to the semi-automated system (Table 2 and Table 3).

## 4. Discussion

Multiple studies demonstrate that laboratory automation may be effective in reducing turnaround time and increasing laboratory productivity [16,17,18]. It may also enhance standardization for accreditation purposes, thereby improving the quality of testing, minimizing manually intensive labor, and strengthening sample management and traceability [17]. This study demonstrated that HLA-B*27 genotyping using fully-automated procedures is an efficient and accurate method compared to semi-automated procedures in the Taiwanese context.

Various types of methods can be employed to detect HLA-B*27, ranging from old, basic, tedious, and time-consuming procedures to rapid throughput techniques. In 1997, Nieto et al. reported the use of polymerase chain reaction with the restriction fragment length polymorphism (PCR-RFLP) for genotyping HLA-B*27. The authors concluded that PCR-RFLP demonstrated robustness, technical simplicity, and cost-effectiveness [19]. PCR-RFLP has also been used to compare the clinical features of AS, along with the frequencies of HLA-B*27 and its alleles in patients from Turkey [20]. However, the main drawbacks of RFLPs are that they are labor intensive and time consuming [21]. Tiemann et al. demonstrated that performing real-time PCR using LightCycler technology is a feasible method for the detection of HLA-B*27, and it is also an alternative to a conventional PCR with sequence-specific primers (PCR-SSP) [22]. Similarly, two studies reported that the use of real-time PCR assays for the detection of HLA-B*27 was fast and reliable in clinical settings [23,24]. Further, real-time PCR has also been applied to detect HLA-B*27 in the Dutch population [25]. Recently, Geiger et al. reported that direct real-time PCR-based HLA-B*27 testing in whole blood was in 100% concordance with the results derived from routine DNA-based HLA-B*27 genotyping [14]. Flow cytometry is also a diagnostic tool used for the detection of HLA-B*27 as it is economical and relatively simple [11,26]. Previously, Skalska et al. demonstrated that the genetic sequence-based method and the flow cytometry method provide consistent results in 99% of samples [27]. However, a limitation of flow cytometry is that it is unable to discriminate HLA-B*27 allelic variants. Priyathersini et al. suggested that flow cytometry is a reliable assay for the detection of HLA-B*27 during the screening of AS in the Indian population [11]. Moreover, Weckel et al. reported that three HLA-B*27-typing kits by flow cytometry are feasible and reliable for diagnosis in 90% of the routine cases [28]. However, another study demonstrated that PCR-SSP has certain advantages over flow cytometry, as it can be carried out with a lower quantity of blood samples, whereas flow cytometry requires more fresh samples for accurate results [29].

DNA extraction has evolved from solution- and solid-phase manual techniques, which were initially performed manually, to incorporate automated methods. The use of high-quantity and high-quality DNA is an important aspect of downstream applications. Therefore, nucleic acid extraction is an important step in laboratory procedures, which are required to carry out further molecular research applications. It is necessary to select a feasible and reliable extraction method, and there are a few things to consider when estimating the available options. These considerations may include cost-effectiveness, technical requirements, time efficiency, and storage requirements [30]. The Maelstrom^TM^ 8 Autostage (Taiwan Advanced Nanotech) utilizes a magnetic-bead-based system to extract nucleic acids. This system is designed to be easily used as it can process up to eight samples simultaneously. Our previous study reported that the Maelstrom^TM^ 8 Autostage offers standardized procedures, the avoidance of sample-to-sample cross contaminations, ease of use, improvement in turnaround time, and lesser hands-on time compared to the manual extraction method (QIAamp Viral RNA Mini Kit) [15].

The fully automated processing of clinical samples results in reduced hands-on time. Such processing also results in high-throughput ability, adequate yield, and purity, which are necessary to achieve routine downstream assay. The LabTurbo^TM^ AIO system has been designed as a fully automated and enclosed instrument to eliminate manual handling. In the present study, we demonstrated that the mean absorbance (optical density) values for the Maelstrom^TM^ 8 and LabTurbo^TM^ AIO systems were 1.88 and 1.9, respectively. Further, the housekeeping gene was amplified during the real-time PCR reaction as an internal control for the extraction method. The mean Ct values for the Roche Cobas z480 and LabTurbo^TM^ AIO systems were 22.7 and 20.4, respectively (Figure 1). Based on our findings, the LabTurbo^TM^ AIO system ensured the isolation of good-quality DNA.

After comparing the results of the semi-automated and fully automated assays, we found that the fully automated assay was superior in detecting HLA-B*27. Further, we found that the use of the LabTurbo^TM^ AIO system shortened the turnaround time by approximately 35.5% while simultaneously handling 48 samples. We required 60 min to load DNA samples and reagents (Pharmigene PG27) on a 96-well plate, whereas we only required 25 min to load DNA samples and reagents (Pharmigene PG27) on a 96-well plate on the LabTurbo^TM^ AIO system.

A major concern in the implementation of a fully automated assay is the potential for the cross-contamination of negative specimens as a consequence of aerosolization, faulty robotics, or robotic errors during the process of nucleic acid extraction for applications in amplification analysis [31]. However, we did not observe any specimen cross-contamination following extraction using the LabTurbo^TM^ AIO system.

As mentioned above, the multiplex RT-PCR implemented on the LabTurbo^TM^ AIO system offered a high-throughput detection of pathogens with a short turnaround time [5]. The LabTurbo^TM^ AIO system utilizes a patented “membrane tube vacuum flow extraction technology” to improve detection sensitivity by producing high-purity and high-yield total nucleic acid [32]. Recently, Jian et al. reported that the LabTurbo^TM^ AIO system can handle 864 samples per day in a continuous operation mode [32]. To the best of our knowledge, the present study was the first to detect HLA-B*27 among the Taiwanese population using the LabTurbo^TM^ AIO system. The main strength of our study is that it evaluates the performance of the LabTurbo^TM^ AIO system for the detection of HLA-B*27, whereas its main drawback is that it did not obtain clinical information about the patients from the physical examination center and, therefore, could not determine the frequency of HLA-B*27 in central Taiwan. Future studies should aim to overcome this limitation by including patients from the outpatient department. 

## 5. Conclusions

In summary, the LabTurbo^TM^ AIO system is a fast and reliable technique for HLA-B*27 detection during routine laboratory testing. We have demonstrated the feasibility and reliability of a fully automated molecular analysis of HLA-B*27 with high throughput and significantly less hands-on time by using the LabTurbo^TM^ AIO system.

## Figures and Tables

**Table 1:** Detected and undetected results on the semi-automated and fully automated assay.

	Semi-Automated		
Fully Automated	Positive	Negative	Total
Positive	20	0	20
Negative	0	30	30
Total	20	30	50

**Table 2:** Sequential flow of manual steps and the time required for each step in the detection of HLA-B*27.

Step	Activity	Type	Complexity	Duration
1	DNA extraction (Maelstrom^TM^ 8)	Semi-automated	Medium	110 min
2	DNA quantification and concentration adjustment (MaestroNano^®^ Pro Spectrophotometer)	Semi-automated	Medium	40 min
3	Load samples and reagents (Pharmigene PG27) on 96-well	Manual	High	60 min
4	Real-time PCR (Z480)	Automated	Low	85 min
5	Test results verification	Manual	Low	15 min

**Table 3:** Sequential flow of automated steps and the time required for each step in the detection of HLA-B*27.

Step	Activity	Type	Complexity	Duration
1	DNA extraction (LabTurbo^TM^ AIO system)	Automated	Medium	80 min
2	DNA quantification and concentration adjustment (MaestroNano^®^ Pro Spectrophotometer)	Semi-automated	Medium	20 min
3	Load samples and reagents (Pharmigene PG27) on 96-well(LabTurbo^TM^ AIO system)	Automated	Low	25 min
4	Real-time PCR (LabTurbo^TM^ AIO system)	Automated	Low	70 min
5	Test results verification (LabTurbo^TM^ AIO system)	Automated	Low	5 min

## Data Availability

Not applicable.

## Supplementary Materials

The following supporting information can be downloaded at: https://www.mdpi.com/article/10.3390/biomedicines11030986/s1, Figure S1: Flow chart of automation.
