# Supplementary figures and images for: High-Throughput and Automated Detection of HLA-B*27 Using the LabTurboTM AIO System
Source: Biomedicines. 2023 Mar 22;11(3):986. doi: 10.3390/biomedicines11030986 (PMC10046602; doi:10.3390/biomedicines11030986)

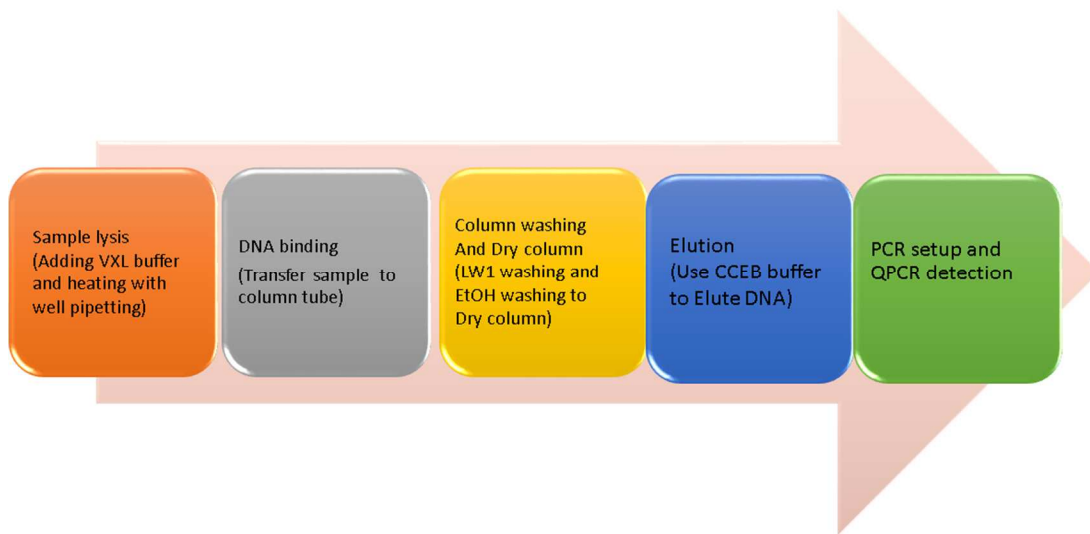

**Figure S1.** Flow chart of automation.

Supplement: Supplementary file 1 [file biomedicines-11-00986-s001.zip › biomedicines-2295373-supplementary.pdf]
